# Fractures in Oxford unicompartmental knee arthroplasty are associated with a decreased medial keel-cortex distance of the tibial implant

**DOI:** 10.1186/s43019-024-00237-2

**Published:** 2024-11-22

**Authors:** Julius Watrinet, Daniel Berger, Philipp Blum, Matthias P. Fabritius, Jörg Arnholdt, Rolf Schipp, Wolfgang Reng, Paul Reidler

**Affiliations:** 1 Department of Orthopaedic Sports Medicine, Technical University Munich, Germany, School of Medicine, Ismaninger Str. 22, 81675 Munich, Germany; 2 Department Trauma Surgery, BG Unfallklinik Murnau, Professor-Küntscher-Straße 8, 82418 Murnau, Germany; 3 Joint Replacement Institute, Klinikum Garmisch-Partenkirchen, Endogap, Auenstraße 6, 82467 Garmisch-Partenkirchen, Germany; 4 Department of Radiology, University Hospital, LMU Munich, Marchioninistr. 15, 81377 Munich, Germany; 5 Department of Orthopedics and Trauma Surgery, Musculoskeletal University Center Munich (MUM), University Hospital, LMU Munich, Marchioninistr. 15, 81377 Munich, Germany

**Keywords:** Unicompartmental knee arthroplasty, Oxford implant, Periprosthetic fracture, Tibial component sizing, Component positioning, Radiographic parameters

## Abstract

**Purpose:**

This retrospective single-center study aimed to investigate incidence and risk factors influencing tibial periprosthetic fractures (TPF) in Oxford unicompartmental knee arthroplasty (UKA), with a specific focus on tibial component positioning and sizing.

**Methods:**

A total of 2063 patients with medial UKA using the Oxford® mobile partial knee implant were analyzed between July 2014 and September 2022. Various preoperative and postoperative radiographic parameters determining pre- and postoperative alignment and implant positioning, incidence and characteristics of periprosthetic fractures, and patient demographics were assessed. Statistical analyses, including Mann–Whitney *U* test and logistic regression, were conducted to identify significant associations and predictors of tibial fractures.

**Results:**

Of the 1853 cases that were finally included in the study, 19 (1%) patients experienced TPF. The fracture group presented with a significantly shorter relative mediolateral and posteroanterior distance between the keel and cortex [mediolateral: 23.3% (23.2–24.8%) versus 27.1% (25.7–28.3%), *p* < 0.001; posteroanterior: 8.4% (6.3–10.3%) versus 10.0% (9.8–10.1%), *p* = 0.004]. Additionally, an increased posterior tibial slope in pre- and postoperative radiographs [preoperative: 10.4° (8.6–11.1°) versus 7.7° (5.4–10.0°), *p* < 0.001; postoperative 9.1° ± 3.1° versus 7.5° (5.9–9.0°), *p* = 0.030] was observed in the fracture group. Furthermore, the use of smaller-sized implants (AA) was associated with higher fracture rates (*p* < 0.001). Anatomical variants, such as a medial overhanging tibial plateau, were not observed.

**Conclusions:**

In UKA, type Oxford TPF are linked to shorter mediolateral and posteroanterior keel-cortex distances, increased pre- and postoperative PTS, and small implant sizes (AA). Fracture lines often extend from the distal keel to the medial tibial cortex. These findings emphasize the importance of precise implant positioning and sizing to minimize fracture risk.

*Level of evidence* Retrospective single-center study, III.

## Introduction

Oxford mobile-bearing unicompartmental knee arthroplasty (UKA) is an established treatment for isolated medial compartmental knee osteoarthritis, demonstrating excellent clinical outcome and long-term survival [1–3]. Tibial periprosthetic fractures (TPF) of the medial tibial plateau after UKA are rare but serious complications associated with increased mortality and morbidity rates [2]. The reported incidence ranges from 3.8% to 8.0% in Asian populations, while in the rest of the world, incidence ranges between 1.2% and 1.6% [4–7]. TPF predominantly occur within the first 3 months after implantation and are atraumatic most of the time [8, 9].

Technical errors [10–14], bony morphology with constitutional overhanging femur condyles and proximal tibia vara, especially in an Asian population [15–17], and the use of undersized implants are reported to be associated with an increased risk of medial TPF [18]. Kamenaga et al. observed a shorter distance of the keel to the posterior cortex to be associated with TPF, which they attributed to malpositioning of the tibial component [19, 20]. The current literature on TPF following UKA is limited by the lack of large, comprehensive patient cohorts, which hampers the ability to fully understand the epidemiology and risk factors associated with this complication [21].

The aim of this study was to evaluate the relationship between the position of the tibial component in frontal and sagittal radiographs and the incidence of TPF. Furthermore, the effects of various factors associated with TPF such as bony morphology, patients demographics, and component size were analyzed. It was hypothesized that TPF are linked to a smaller mediolateral keel-cortex distance, especially in small implants.

## Materials and methods

**Fig. 1:**
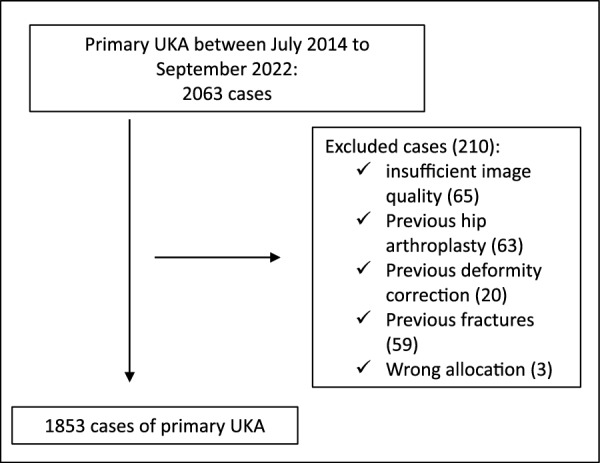
Flowchart showing the patient selection process

This retrospective, single-center study was approved by the Institutional Review Board and conducted in accordance with the Declaration of Helsinki (no. 22-0990 KB). Requirement for written informed consent was waived, and all cases were analyzed anonymously. Between July 2014 and September 2022, 2063 patients were treated with medial UKA using the Oxford® mobile partial knee implant (Biomet, Warsaw, IN, USA) and included in this study. However, 210 (10.2%) patients were excluded from the study due to insufficient image quality, previous hip arthroplasty, previous deformity correction, previous fractures, or wrong allocation (Fig. 1).

Indication for UKA in cases of symptomatic isolated medial knee osteoarthritis met the patient selection criteria of Hamilton et al. [22]. Press-fit fixation was the standard procedure for implant placement and only mobile-bearing was used. Cemented implant placement was chosen individually in cases with a high likelihood of poor bone quality, considering risk factors such as age, sex, and concurrent health conditions. The occurrence of fractures was determined from medical records on follow-ups. Fractures were classified accordingly to Burger et al. [8]: fracture lines from tibial resection to medial cortex, causing large (I) or small (II) medial plateau fractures; (III) varus/anterior subsidence with a small medial fragment; (IV) posteromedial plateau fracture from screw fixation; (V) fracture from tibial keel to medial cortex; and (VI) bicondylar plateau fracture with lines to medial/lateral cortex.

All UKAs were performed either by or under the supervision of a senior consultant at a certified arthroplasty center, according to the manufacturer's instructions as previously described [23]. The rehabilitation protocol allowed immediate, pain-dependent full weight bearing.

Preoperatively, extended radiographs of the knee in two planes were obtained within 1 month prior to surgery as well as anteroposterior (a.p.) weight-bearing radiographs when available. Postoperative two-plane radiographs of the knee were obtained within the first week after surgery. The radiographs were evaluated using JiveX Review 5.4.0.6 software (VISUS Health IT GmbH, Bochum, Germany). Radiographic measurements were conducted by a single rater. To assess intra- and interrater reliability, two raters evaluated a subgroup of 40 participants three times, with 6-week intervals between each evaluation.

**Fig. 2:**
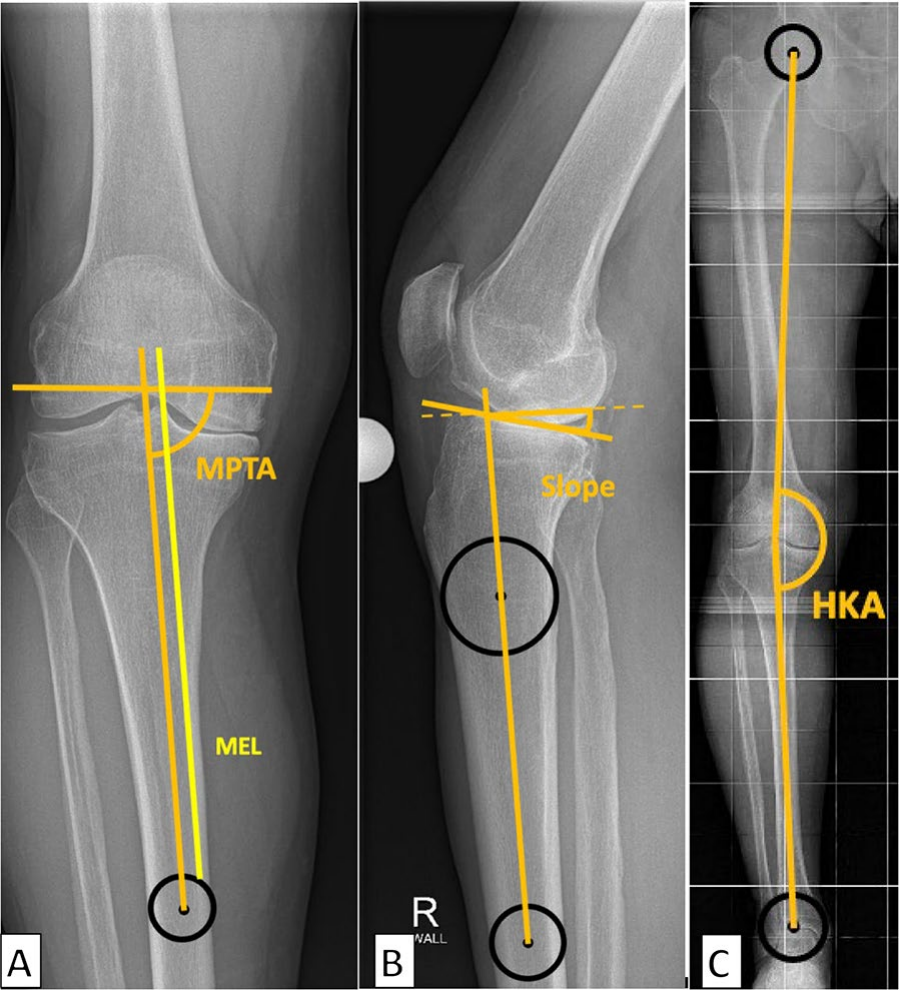
Preoperative measurements: measurement of the mechanical proximal tibia angle and the medial eminence line (MEL), (**A**) measurement of the sagittal posterior slope of the medial tibiaplateau, (**B**) measurement of the hip-knee-ankle angle (HKA) (**C**)

The analyzed parameters included lower limb alignment assessed on extended knee radiographs (Fig. 2A, B) and long-leg radiographs (Fig. 2C). In the a.p. projection, the tibial mechanical axis (mT) was defined as a line passing through the center of the distal tibia and the center of the intercondylar region. The mechanical medial proximal tibial angle (MPTA) was defined as the angle between the mT and a line drawn parallel to the articular surface of the proximal tibia. The medial eminence line (MEL, yellow line) was drawn as a line passing through the apex of the medial intercondylar eminence, parallel to the tibial mechanical axis. (Fig. 2A). In the lateral projection, the mT was defined as a line passing through the center of the tibia at the tuberositas and the visible distal end of the tibia. The posterior tibial slope (PTS) was measured for the medial tibial plateau as the angle between an orthogonal to the mT and a parallel to the medial tibial plateau (Fig. 2B). The hip-knee-ankle angle (HKA) was measured on preoperative full-leg radiographs [24] (Fig. 2C).

### Postoperative measurements

**Fig. 3:**
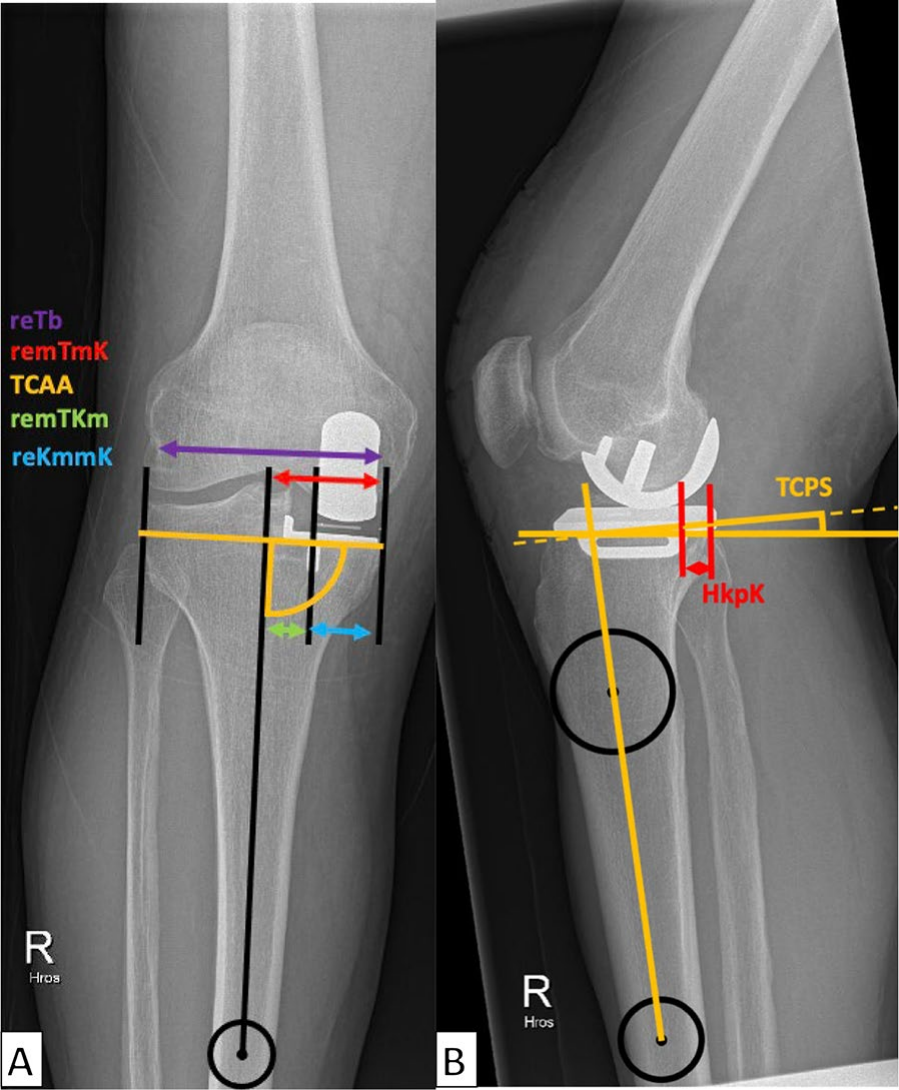
Postoperative measurements. X-ray was taken in a weight-bearing situation: width of the tibiaplateau (reTb, violet), medial tibiaplateau (remTmK, red), tibial component alignment angle (TCAA, yellow), distance from mechanical tibial axis to implant keel (remTKm, green), and distance from keel to medial tibial cortex (reKmmK, blue) were measured (**A**); tibial component sagittal posterior slope (TCPS, yellow) and distance from keel to posterior cortex (HkpK, red) at resection level were obtained (**B**)

In the postoperative a.p. radiograph, the distances from the mT to the medial cortex (remTmK, red) and the keel center (remTKm, green) were determined. The proximal tibial width (reTb, violet) was defined as the distance between the most medial and lateral tibial plateau. Additionally, the distance between the keel and the medial cortex (reKmmK, blue) was determined by subtracting remTKm from remTmK. The tibial component alignment angle (TCAA) was measured as previously described (Fig. 3A) [25]. In the lateral projection, the postoperative tibial component posterior slope (TCPS) of the implant was measured as the angle between an orthogonal to the mechanical sagittal tibia axis and a parallel to the implant. Additionally, the distance between the posterior keel and the posterior cortex (HkpK) was measured at the resection level (Fig. 3B). The lateral postoperative image was calibrated using the narrower part of the femoral implant stem, which had a consistent diameter of 6 mm for all implant sizes.

### Data analyses

R-Studio (version 2023.01, posit, Boston, MA, USA) was used for statistical analysis. *p*-Values less than 0.05 were considered statistically significant. Patients were grouped on the basis of the presence or absence of a TPF. Hence, data were not normally distributed according to the Kolmogorov–Smirnov test, and median and interquartile range were calculated and are represented as median (IQR). The Mann–Whitney *U* test was used to compare the characteristics of the two groups. The Wilcoxon test was used to compare pre- and postoperative values. Pearson’s chi-squared test was used to assess whether differences in categorical data were significant. Associations between fracture risk and confounding factors were evaluated using univariate logistic regression analyses for conformation of associations. Multiple logistic regression analyzed the association of reKmmK to TPF with respect to confounder (HkpK, PTS, TCPS, female sex, age, tibial component size AA). Odds ratios (OR) including 95% confidence intervals and* p*-values were calculated. Intra- and interrater reliabilities of each radiographic parameter were assessed by calculating the intraclass correlation coefficient (ICC).

Post hoc power analysis was performed using G* Power 3 [26]. With a type-I error (*α*) of 0.05 and a total sample size of 1853, the study provided a power (1-β) of greater than 0.99 for detecting an effect size q (H1) of 1.5, calculated on the basis of the means and standard deviation for reKmmK in both groups.

## Results

Out of the 1853 patients included, 19 (1.0%) experienced a TPF, while the remaining 1834 patients were included in the non-fracture group. The mean follow-up duration was 5.1 ± 2.4 years, with a minimum follow-up period of 1 year.

Demographic and preoperative data for the two groups are presented in Table 1. Significant differences were observed in age distribution between the groups (67.2 versus 74.1 years; *p* = 0.001). Additionally, the fracture group had a higher percentage of female patients (78.9%) compared with the non-fracture group (47.2%) (*p* = 0.001). Both intra- and interrater reliabilities were excellent for radiographic measurement according to the ICCs (Tables 2 and 3).

**Table 1:** Preoperative demographic data

	Non-fracture	Fracture	*p*-Value
Number of cases (*n*)	1834	19	
Age (years)	68 (61–74)	74 (71–78)	0.001
Female sex (*n*/%)	867 (47.2%)	15 (78.9%)	0.001
BMI (kg/m^2^)	28 (25–31)	28 (23.5–32.5)	0.904
Follow-up (years)	5.3 (3.2–7.1)	5.4 (2.5–7.3)	0.684
Cemented implants (*n*/%)	336 (18.3%)	2 (10.5%)	0.382
MEL (*n*)	8 (0.4%)	0 (0%)	0.775

Values are displayed as medians with interquartile ranges (Q1 25%–Q3 75%). The data were tested using the Mann–Whitney *U* test to compare differences between groups.* MEL* medial eminence line, *BMI* body mass index

**Table 2:** Preoperative measurement of the leg alignment and the ICCa

	Non-fracture (*n* = 1834)	Fracture (*n* = 19)	*p*-Value	ICC (95%)	
				Intrarater ICC	Interrater ICC
HKA [°]	173.6 (171.4–176.0)*	172.2 (171.2–173.9) **	0.279	0.96 (0.94–0.98)	0.96 (0.93–0.98)
MPTA [°]	86.4 (85.1–87.6)	86.6 (84.9–87.7)	0.857	0.93 (0.89–0.96)	0.87 (0.76–0.93)
PTS [°]	7.7 (5.4–10.0)	10.4 (8.6–11.1)	< 0.001	0.94 (0.91–0.97)	0.94 (0.89–0.97)

Values are displayed as medians with interquartile ranges (Q1 25%–Q3 75%). The data were tested using the Mann–Whitney *U* test to compare differences between groups

*HKA*  hip-knee-ankle angle, *MPTA*  medial proximal tibia angle, *PTS*  posterior tibial slope, *CI * confidence interval

*Available in 1257 cases

**Available in 12 cases

**Table 3:** Postoperative measurement of the tibial anatomy and the ICCa

	Non-fracture (*n* = 1834)	Fracture (*n* = 19)	*p*-Value	ICC (95%)	
				Intrarater ICC	Interrater ICC
reKmmK [%]	27.1 (25.7–28.3)	23.3 (23.2–24.8)	< 0.001	0.96 (0.93–0.98)	0.90 (0.82–0.95)
remTmK [%]	49.2 (48.1–50.2)	48.8 (47.7–49.8)	0.400	0.95 (0.91–0.97)	0.65 (0.33–0.81)
remTKm [%]	22.1 (20.6–23.5)	24.5 (23.6–26.7)	< 0.001	0.91 (0.84–0.95)	0.84 (0.71–0.92)
HkpK [%]	10.0 (9.8–10.1)	8.4 (6.3–10.3)	0.004	0.98 (0.97–0.99)	0.98 (0.96–0.99)
TCAA [°]	88.5 (86.9–89.9)	89.0 (87.4–91.0)	0.193	0.93 (0.88–0.96)	0.93 (0.87–0.96)
TCPS [°]	7.5 (5.9–9.0)	9.0 (7.3–11.0)	0.030	0.94 (0.91–0.97)	0.92 (0.86–0.96)

Values are displayed as medians with interquartile ranges (Q1 25%–Q3 75%). The data were tested using the Mann–Whitney *U* test to compare differences between groups

*reKmmK*  distance from the implant keel to the medial tibial cortex, *remTmK*   distance from the mechanical tibial axis to the medial tibial cortex, *remTKm* distance from the mechanical tibial axis to the implant keel, *HkpK* distance from the posterior keel to the posterior cortex, *TCAA* tibial component alignment angle, *TCPS* tibial component posterior slope, *CI* confidence interval

**Fig. 4:**
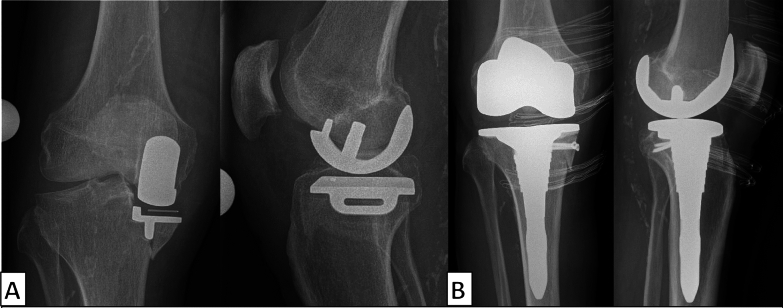
Tibial periprosthetic fracture Burger type V (**A**). Conversion to total knee arthroplasty with a tibial stem and two single screw fixation of the medial plateau (**B**)

**Fig. 5:**
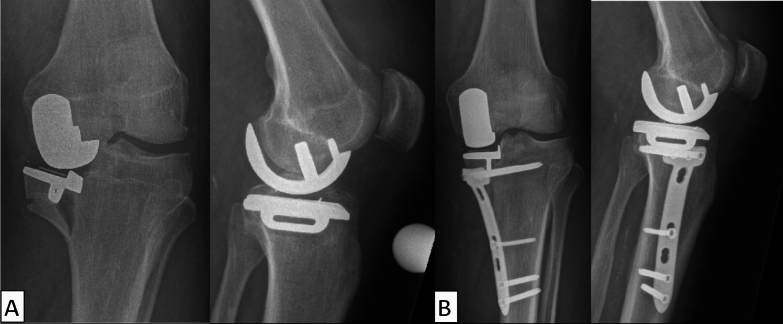
Tibial periprosthetic fracture Burger type V (**A**). Implant preserving open reduction and internal fixation (ORIF) (**B**)

The fractures occurred 66 days (range 15–220 days) after surgery, and no intraoperative fractures were observed. Fracture lines were classified according to Burger et al. and type V was present in 16 cases, while type IV-like fractures were observed in 2 cases and type I was documented in 1 case [8]. A total of 12 patients underwent open reduction and internal fixation (ORIF) and 7 underwent conversion to total knee arthroplasty (Figs. 4 and 5).

Known risk factors, AA-sized tibial implants had higher fracture rates (10.9% versus 0.7%) (Table 4). Multivariate analysis identified reKmmK, PTS, age, and AA size as independent risk factors (Table 5). Moreover, the presence of an overhanging tibial plateau was not evident in the study population and MEL showed no significant difference (*p* = 0.77).

**Table 4:** Overview of the distribution of tibial component sizes among patients with tibial plateau fractures (TPF)

Tibial component size	AA	A	B	C	D	E	F
Total [*n*]	64	254	350	465	457	217	46
Fractures [*n*]	7	2	6	4	0	0	0
Fracture rate [%]	10.9%	0.8%	1.7%	0.9%	0.0%	0.0%	0.0%

**Table 5:** Factors associated with periprosthetic fractures

	Univariate analysis		Multivariate analysis	
	Odds ratio	*p*-Value	Odds ratio	*p*-Value
reKmmK	0.58 (0.49–0.71)	< 0.001	0.60 (0.48–0.72)	< 0.001
HkpK	0.72 (0.58–0.90)	< 0.001	0.82 (0.64–1.01)	0.079
PTS	0.8 (0.70–0.91)	< 0.001	0.78 (0.66–0.92)	0.003
TCPS	0.77 (0.65–0.92)	< 0.001	0.84 (0.69–1.02)	0.075
Female sex	4.21 (1.39–12.73)	< 0.001	2.86 (0.91–11.38)	0.095
Age	1.09 (1.03–1.16)	< 0.001	1.09 (1.03–1.17)	0.007
Tibial component size AA	20.1 (7.4–54.8)	< 0.001	5.67 (1.54–20.40)	0.008

Variables were initially tested using a univariate analysis within a regression model to confirm their associations. Upon confirmation, all factors were subsequently included in a multivariate regression model to identify independent associations. Odds ratio is displayed with 95% confidence interval. The data were tested using the univariate and multivariate logistic regression

*reKmmK* distance from the implant keel to the medial tibial cortex, *HkpK* distance from the posterior keel to the posterior cortex, *PTS* posterior tibial slope, *TCPS* tibial component posterior slope

Preoperative leg alignment measurements, including HKA, MPTA, and PTS, are presented in Table 2. The fracture group had a higher preoperative PTS (10.4° versus 7.7°, *p* < 0.001). Postoperatively, reKmmK differed significantly (fracture group: 27.1% versus non-fracture: 23.3%, *p* < 0.001, odds ratio 0.58, corrected 0.60; Table 3). HkpK also varied (*p* = 0.004), with reKmmK and HkpK showing a positive correlation (*r* = 0.047, *p* = 0.042). TCPS differences were significant (*p* = 0.03).

## Discussion

The key finding of this study was that the mediolateral and posteroanterior distance between implant keel and tibial cortex is reduced in patients who experienced fractures over time. A far medial implant position was associated with a shorter distance to the posterior cortex, thereby influencing the risk of TPF. In Oxford UKA, most TPF extend from the distal pole of the keel to the medial cortex of the tibia (Burger type V). Fractures are further linked to reduced bony support of the implant by malpositioning.

There is limited evidence to suggest that TPF in UKA are associated with implant position. However, a shorter distance from the tibial component keel to the posterior cortex (HkpK) has also been correlated with a higher risk of fracture, which supports the present findings [19]. Although this study found a significant association between increased PTS and TPF, there are no other reports on an association between PTS and the occurrence of fractures. From a biomechanical perspective, increased PTS and TCPS are associated with higher contact stress on the tibial plateau, which can weaken the bone structure and increase the risk of TPF [27, 28].

In the present study, tibial implant size AA was highly associated with TPF compared with other sizes, which is consistent with the results of other studies and illustrates that associate tibial implant size is a confounding factor [18, 19]. Additionally, reKmmK was significantly smaller in size AA tibial components, suggesting less support to the tibial bone stock and might be associated to an undersizing of the tibial component [20]. The lack of supporting bone stock in smaller tibial plateaus during keel preparation or insertion is discussed as one potential mechanism contributing to the increased fracture risk [18]. Favorable outcomes after medial UKA are associated with varus alignment [29–31], as valgus alignment correlates with reduced supporting bone mass [19]. Despite these radiologic findings, the reason for the increased fracture risk associated with small components remains unclear.

Anatomical variants such as a medial overhanging tibial plateau were not observed in our study sample and diverged compared with Asian populations, indicating that they be a distinct subgroup. Consequently, the radiographs revealed a different fracture pattern and frequency in this study group compared with the results from similar studies conducted in Asia [7, 32].

Fracture risk increases with age and with female sex [33]. In the present study, higher age was significantly associated with TPF, and there was a tendency toward an increased fracture risk in female patients. This aligns with existing literature, where Wood et al. identified age and sex as a significant risk factor, and Burger et al. reported that advanced age (*p* = 0.003) was a risk factor for TPF following UKA [8, 21].

## Limitations

First, the retrospective design inherently introduces a risk of selection bias. Although rotational and projection errors in radiographs may present limitations to this study, evidence persists regarding the influence of mediolateral implant positioning on fracture risks [27]. Although different surgeons performed the procedures, all were highly trained, and the large number of cases indicates a high level of skill and comparability. A limitation is that 316 (16%) of patients had a 1–2-year follow-up. However, since Burger et al. reported 90% of fractures within the first year, this impact may be minimal [8]. Additionally, the radiographic evaluations were conducted by a single rater; however, in a subset of cases, we observed excellent inter- and intrarater reliability. Further limitations include the incomplete availability of long-leg radiographs, no bone mineral density measurement, and the retrospective nature of the study. To best of our knowledge, we have presented the largest study sample of TPF after UKA Type Oxford implantation to date.

These findings emphasize the importance of prioritizing implant positioning especially concerning the keel-cortex distance and tibial slope, which should be considered during planning and implantation. This study’s strengths lie in its large cohort size and detailed radiographic analysis, which allowed for the identification of specific risk factors influencing fracture risk. To understand how implant positioning impacts fractures, further biomechanical investigations are imperative. In addition, these insights provide valuable information about bony support to refine future prosthetic designs and achieve optimal implant placement.

## Conclusions

In UKA, Type Oxford TPF are linked to shorter mediolateral and posteroanterior keel-cortex distances, increased pre- and postoperative PTS, and small implant sizes (AA). Fracture lines often extend from the distal keel to the medial tibial cortex. These findings emphasize the importance of precise implant positioning and sizing to minimize fracture risk.

## Data Availability

Data cannot be made available.
